# Describing vegetation characteristics used by two rare forest-dwelling species: Will established reserves provide for coastal marten in Oregon?

**DOI:** 10.1371/journal.pone.0210865

**Published:** 2019-01-31

**Authors:** Katie M. Moriarty, Jake Verschuyl, Andrew J. Kroll, Raymond Davis, Joshua Chapman, Bruce Hollen

**Affiliations:** 1 USDA Forest Service, Pacific Northwest Research Station, Olympia, Washington, United States of America; 2 National Council for Air and Stream Improvement, Western Sustainable Forestry Program, Anacortes, Washington, United States of America; 3 Weyerhaeuser, Springfield, Oregon, United States of America; 4 USDA Forest Service, Region 6, Corvallis, Oregon, United States of America; 5 USDA Forest Service, Region 6 Regional Office, Portland, Oregon, United States of America; 6 USDI Bureau of Land Management, Regional Office, Portland, Oregon, United States of America; University of Maine, UNITED STATES

## Abstract

Forest management guidelines for rare or declining species in the Pacific Northwest, USA, include both late successional reserves and specific vegetation management criteria. However, whether current management practices for well-studied species such as northern spotted owls (*Strix occidentallis caurina*) can aid in conserving a lesser known subspecies—Humboldt martens (*Martes caurina humboldtensis*)–is unclear. To address the lack of information for martens in coastal Oregon, USA, we quantified vegetation characteristics at locations used by Humboldt martens and spotted owls in two regions (central and southern coast) and at two spatial scales (the site level summarizing extensive vegetation surveys and regionally using remotely sensed vegetation and estimated habitat models). We estimated amount of predicted habitat for both species in established reserves. If predicted overlap in established reserves was low, then we reported vegetation characteristics to inform potential locations for reserves or management opportunities. In the Central Coast, very little overlap existed in vegetation characteristics between Humboldt martens and spotted owls at either the site or regional level. Humboldt martens occurred in young forests composed of small diameter trees with few snags or downed logs. Humboldt martens were also found in areas with very dense vegetation when overstory canopy and shrub cover percentages were combined. In the South Coast, Humboldt martens occurred in forests with smaller diameter trees than spotted owl sites on average. Coastal Humboldt martens may use stands of predicted high quality spotted owl habitat in the Pacific Northwest. Nonetheless, our observations suggest that coastal Humboldt martens exist in areas that include a much higher diversity of conifer size classes as long as extensive dense shrub cover, predominantly in the form of high salal and evergreen huckleberry, are available. We suggest that managers consider how structural characteristics (e.g., downed logs, shrub cover, patch size), are associated with long-term species persistence rather than relying on reserves based on broad cover types. Describing vegetation may partially describe suitability, but available prey or predation risk ultimately influence likelihood of individual Humboldt marten use. Guidelines for diversifying vegetation management, and retaining or restoring appropriate habitat conditions at both the stand level and regionally, may increase management flexibility and identify forest conditions that support both spotted owls and Humboldt martens.

## Introduction

The “umbrella species” concept posits that habitat conservation for one species with certain overarching characteristics of behavior and habitat use with larger home ranges will provide habitat for species with similar, or more narrow, niches [1, 2]. Accurate descriptions of habitat conditions for a target (or well-studied) species can guide management activity but often information is lacking for rare or little-known species [3]. Assumptions underlying the umbrella species concept must be validated, and over-simplistic or qualitative descriptions of habitat conditions (e.g., old growth forest) for both the umbrella and other species can result in management that does not provide attributes necessary for persistence or recovery of lesser known species (e.g., rare species recovery, [4]).

Northern spotted owls (*Strix occidentallis caurina*) and Pacific martens (*Martes caurina*) are both associated with late-successional forests with high canopy cover, often in stands with trees having complex structures, such as branch whorls, cavities, hollows, or chambers, that provide locations for denning and nesting [5, 6]. Late-successional reserves have been designated for northern spotted owls in coastal Oregon, USA [7–9]. When reserves were designated, little was known about coastal Oregon populations of Pacific martens, which are now recognized as the subspecies, Humboldt marten (*Martes caurina humboldtensis*). Unlike most marten populations in North America, this subspecies, occupies coastal forests with limited or no snow cover. Coastal marten populations have contracted in the 20^th^ century [10], prompting petitions and a proposal to list Humboldt martens as Federally threatened [11, 12]. If Humboldt martens occupy similar areas as northern spotted owls, then the current reserve allocation in Oregon may be adequate for retaining Humboldt marten populations.

The northern spotted owl figures prominently in forest management discussions in coastal Oregon [9, 13, 14], and recently Humboldt martens have been the focus of specific management recommendations. Northern spotted owls and Humboldt martens are similarly sized predators (600-1000g) with life history traits characteristic of k-selected species (e.g., age of first reproduction is around 2 years). Both spotted owls and Humboldt martens require access to prey, and may be negatively affected by competition and predation, including interactions with barred owls *(Strix varia*) [15–17]. Northern spotted owls were listed as a threatened subspecies in 1990 under the federal Endangered Species Act [18]. Humboldt martens in coastal Oregon and California were petitioned for listing in 2010 [11]. The U.S. Fish and Wildlife Service determined that a coastal Distinct Population Segment of the Pacific marten, including the Humboldt marten, did not warrant listing as a federally threatened or endangered under the Endangered Species Act in 2015 [19], but recent litigation required them to re-evaluate the petition [20, 21]. Most recently, the U.S. Fish and Wildlife Service provided a Proposed Rule for martens to be considered as Threatened [12]. A summary of the assessment is available [21], but the conservation strategy primarily focuses on California where more information is known [22] and where Humboldt martens have recently been listed as State Endangered [23]. Martens are also a U.S. Forest Service Sensitive Species on three National Forests in Oregon, a Forest Service Management Indicator Species on most National Forests in Oregon, and an Oregon Conservation Strategy Species [24].

We evaluated the extent to which Humboldt marten and northern spotted owl current use overlapped to determine if spotted owls were a viable umbrella species for martens. To meet these objectives, used a multi-scale approach to (1) provide more detailed information on vegetation characteristics associated with Humboldt marten presence, (2) compare vegetation characteristics at locations used by Humboldt martens with those used by northern spotted owls, and (3) evaluate the amount of predicted Humboldt marten habitat within current established reserves, by analyzing vegetation data at two spatial scales. First, we collected fine-scale vegetation data to describe the range of vegetative characteristics used by either Humboldt martens or spotted owls within three coastal regions of Oregon (Humboldt marten rest sites in the Central Coast, spotted owl telemetry near Coos Bay, and Humboldt marten locations obtained with scent detection dog teams in the South Coast). Second, within 10 km of the contemporary marten population boundaries, we paired our combined Humboldt marten surveys and data collected for spotted owl demographic research with remotely sensed vegetation data to create coarse-scale suitability models in the Central and South Coast regions. To examine efficacy of using areas designated as Late Successional Reserves for spotted owls for the Humboldt marten, we examined whether predicted Humboldt marten distribution was included within established late-successional reserves designed to protect the spotted owl [25]. Finally, we describe conditions for fine- and broad-scale vegetation restoration activities to increase the probability of Humboldt marten occurrence near extant populations.

## Materials and methods

**Fig 1:**
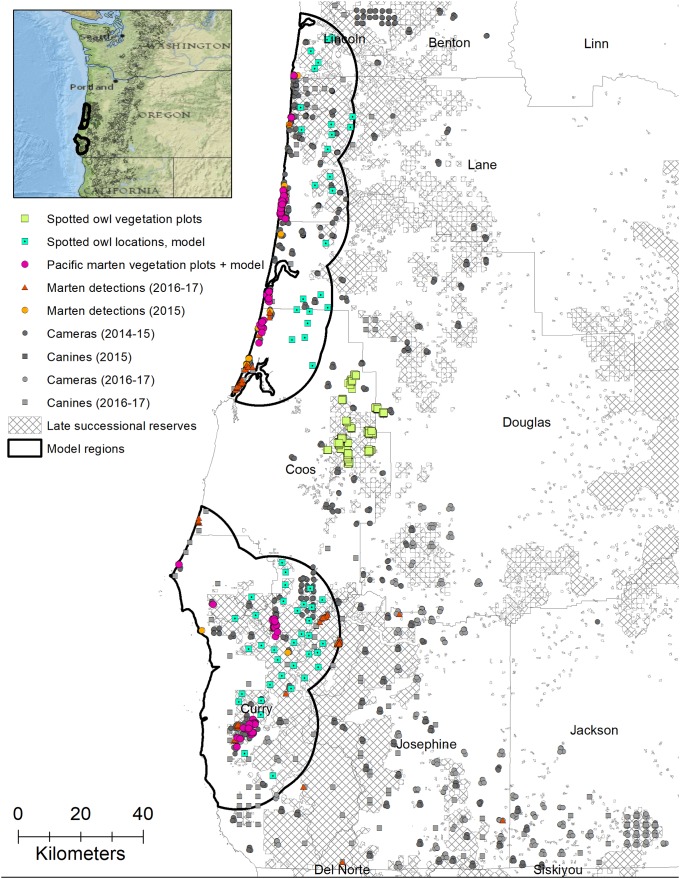
Study area. Survey and modeling regions to compare northern spotted owl (*Strix occidentalis caurina*) and coastal Pacific marten (*Martes caurina humboldtensis*) predicted habitat. We radio-tagged northern spotted owls and collected data in the core of their range (yellow squares). Both spotted owl (blue squares with a dot) and Humboldt marten distribution surveys (pink circles) were used for modeling predicted distributions, with the extent bounded within 10 km of all Humboldt marten locations. We included more recent marten survey effort (dark grey) and detections (red triangles) to represent surveyed areas were consistent with marten distribution. Cross-hatched areas were designated as late-successional forest reserves under the 1994 Northwest Forest Plan. Imagery sources: Esri, DigitalGlobe, GeoEye, Earthstar Geographics, CNES/Airbus DS, USDA, USGS, AeroGRID, IGN, and the GIS User Community.

We surveyed for Humboldt martens throughout coastal Oregon with the highest sampling densities in areas where Humboldt martens had been previously detected [10, 26]. Our contemporary surveys suggest two populations of Humboldt martens exist in the South and Central Coast [26]. An ongoing northern spotted owl telemetry study occurred adjacent to and between these populations. Thus, for this analysis, we had two Humboldt marten study areas: South (42.5°N, -124.2°W) and Central Coast (43.9°N, -124.1°W) and one spotted owl study area near Coos Bay (43.3°N, -123.9°W) (Fig 1). The maritime climate in all study areas was characterized by cool dry summers (average low and high July temperature = 12, 18°C, 6–9% total precipitation) and mild, wet, winters (average low and high January temperature = 4, 12°C, averages of 100–300 cm of annual precipitation) interspersed with fog and cloud cover year round [27].

The South Coast study area included dominant vegetation on non-serpentine soils of Sitka spruce (*Picea sitchensis*), western hemlock (*Tsuga heterophylla*), Douglas-fir (*Pseudotsuga menziesii*), and mixed conifers near the California border (Franklin and Dyrness 1973). Areas with serpentine soils that supported unique plant communities were interspersed, including forest composed of widely-spaced pine (*Pinus spp*.*)* and an understory of grasses and more mesic areas with a dense and diverse shrub layer including tan oak (*Notholithocarpus densiflorus var*. *echinoides*) and huckleberries (*Vaccinium spp*.) [28, 29]. Hardwoods were rare in this study site. Sampling locations occurred from sea level (0m) to 1304 m.

Vegetation characteristics were similar in the northern spotted owl study area, 51 km north northeast of the South Coast Humboldt marten locations. The spotted owl study area encompassed portions of the Coos Bay BLM District and interspersed private timberlands. Vegetation and climatic conditions in the Coos Bay spotted owl study area were similar to those in the non-serpentine portions of the South Coast with overstory vegetation dominated by western hemlock and Douglas-fir. Hardwoods were rare to absent in this study site. Sampling locations ranged in elevation from 24 to 844 m.

In the Central Coast study area (23 km northwest of the spotted owl study area) we generally found Humboldt martens using dune forests within 1-km of the Pacific Ocean [26]. This area was designated as a recreation area for off road vehicles. Vegetation established on sandy soils was composed predominantly of lodgepole pine (*Pinus contorta var*. *contorta*) with reed grasses (e.g., *Onupta sp*), and waxmertyle (*Myrica californica*) on seasonally flooded sites or mixed Douglas-fir and Sitka spruce with a dense shrub layer dominated by salal (*Gaultheria shallon*) and evergreen huckleberry (*Vaccinium ovatum*) on dry sites [30]. Hardwoods were absent from this study site. Sampling locations ranged in elevation from sea level (0m) to 80m.

To address our first objective of describing fine-scale vegetation characteristics at locations used by Humboldt martens and spotted owls, we compiled locations that animals used with differing survey techniques. We identified vegetation plot locations at (1) high use areas (50% core areas) from spotted owls’ home ranges in the Coos Bay study area, (2) Humboldt martens’ rest sites and telemetry locations in the Central Coast study area, and (3) locations from scent detection dog surveys in the South Coast study area because telemetry data were not available there. Vegetation plots in the Central Coast Humboldt marten area included locations used for resting, and are presumed to be necessary for daily survival [31]. Locations in the South Coast Humboldt marten and Coos Bay spotted owl study areas likely included both foraging and resting or roosting sites.

### Northern spotted owl telemetry

We conducted call-surveys for northern spotted owls, capturing and radio-tracking individuals as possible. Spotted owls were fitted with Holohil RI-2C transmitters with a 12-month battery (10mm x 32mm) weighing 6 grams (Holohil Systems Ltd., Carp, Ontario, Canada). We estimated the spotted owl’s triangulated location using Location of a Signal software (Ecological Software Solutions LLC) and discarded locations with >5 ha (40m radius) error telemetry polygons. To assess triangulation accuracy, radui transmitters were hung at different points on a hillslope (top, middle, lower). Each field biologist was unaware of radio transmitter locations and obtained average location errors <80m. We created 50 percent fixed-kernel core areas using program BIOTAS (Ecological Software Solutions LLC, http://www.ecostats.com/web/Biotas).

We located 23 northern spotted owls and radio-tracked 15 in 9 different portions of the study area. We estimated home ranges of 13 spotted owls with >30 independent locations. Each of these spotted owls was tracked for an average of 14.6 months (range 4–28 months). Triangulated telemetry locations were obtained at night only and teams averaged 1.7 locations recorded per bird per week (*x* = 107 total locations/individual; range: 32–221).

### Humboldt marten rest site locations (Central Coast)

We captured Humboldt martens from October to December 2015 for radio collar attachment and marking using modified live traps (Tomahawk Model 104, Hazelhurst, Wisconsin, USA) with a custom-built wooden cubby at the rear of the trap to provide protection against weather and disturbance. We baited traps with a combination of chicken (~20g), bacon (1/2 strip), organic strawberry jam (~2 oz), and an olfactory lure (Gusto, Minnesota Trapline Products, Pennock, Minnesota, USA). We anesthetized martens intramuscularly with 18mg/kg ketamine hydrochloride (Ketaset 100 mg/ml, Fort Dodge Animal Health, Fort Dodge, Iowa, USA) combined with 0.2 mg/kg midazolam hydrochloride (Versed 5 mg/ml, Bedford Laboratories, Bedford, Ohio, USA) using tested protocols [32]. We radio-collared martens we suspected were >2 years old with a VHF (M1820 27g, Advanced Telemetry Systems, Isanti, Minnesota, USA) or a combined GPS/VHF collar (G10 28g, Advanced Telemetry Systems, Isanti, Minnesota, USA or MicroMini Quantum 4000 40g, Telemetry Solutions, Concord, California, USA) weighing <5% of the individual marten’s body weight. We later verified estimated age of individuals using cementum annuli techniques [33].

We estimated the location of each individual Humboldt marten using VHF radio-telemetry at least twice a week and opportunistically radio-tracked inactive martens to rest sites. We marked each rest site with flagging and tree tags, and took detailed notes of the precise location to ensure we could relocate it for collecting fine-scale vegetation data when the marten was no longer present. Further, we identified areas where we presumed the marten was resting by field observers locating several scats near a hollow or cavity. Often it was difficult to locate the exact position due to the field observer moving through dense brush, scaring the resting marten. Nonetheless, scat position was consistently in the area with the previous radio signal and because we averaged vegetation characteristics within 4 plots (details below), we feel these locations provided data for marten use. We collected fine scale GPS data (locations programmed every 5 minutes) intermittently [34], and used Local Convex Hulls [35] to verify that >90% of rest locations were within the marten’s 75% isopleth.

We collected telemetry data on 7 female and 4 male Humboldt martens in the Central Coast (September 2015–April 2016), acquiring 8103 locations [34] and found 53 resting locations (methods within [36]). A recent population estimate indicated 51–87 individuals [34], suggesting we monitored 11–20% of the total estimated population.

### Humboldt marten locations via scent detection dogs (South Coast)

To identify Humboldt marten locations in the South Coast, we surveyed with scent detection dog teams from University of Washington’s Center for Conservation Biology’s Conservation Canines program. Scent detection teams consisted of a handler and a dog with at least 480 hours of training using lab and simulated field trails (e.g., hidden scats on training boards and in the duff). Detection dog team surveys occurred near recent records of Humboldt marten locations identified using a stratified random design (see [37] for methods). During winter 2015, field teams surveyed 74 randomly located sample units with 2 remote cameras and detected Humboldt martens at 11 locations [26, 37]. Detection dog teams conducted time- and area-constrained search of 1 km^2^ areas centered around all 11 remote camera detections and 11 random remote camera locations without prior Humboldt marten detections. Surveys occurred for at least 6 hours within each area and dog teams were unaware as to whether a Humboldt marten was detected prior. We assumed this level of effort would allow adequate effort to locate scats, especially in areas that Humboldt martens used more frequently [37]. We assume this technique identified a mixture of resting and foraging locations since latrines were often located at martens’ resting areas. We acknowledge scats and remote camera detections could have occurred on the perimeter of martens’ ranges and their core areas, potentially increasing the variability of observed vegetation characteristics. As such, our data for this classification are more likely to represent “foraging” as opposed to “denning and resting” vegetation.

Scat samples were sent to the US Forest Service National Genomics Laboratory (Missoula, MT) for genetic analyses. Whole genomic DNA was extracted samples using the QIAGEN Dneasy Tissue Kit (Qiagen, Valencia, CA, USA). Samples were tested for species identification using the control region of mitochondrial DNA (mtDNA) using universal primers [38]. Samples were analyzed 13 microsatellite loci used in previous studies of mustelids [39–43]. We accepted data from hair samples as error-free only if the microsatellites produced consistent scores using a multi-tube approach [44, 45]. Data was checked for genotyping errors using program Dropout [46]. The samples were also tested using an SRX/SRY analysis to determine sex [47]. Genetic samples were stored at the National Genomics Laboratory. We only conducted vegetation surveys at Humboldt marten locations where scats confirmed genetically to be martens.

### Fine-scale vegetation surveys and analyses

We collected vegetation data identically in each study area using both a 16-m radius fixed plot (0.08 ha, 1/5 acre) and a variable-density estimation of basal area (m^2^/ha). Within each plot, we collected data on snags, shrubs, and downed woody material within each 0.08 ha fixed plot. For all snags we identified species when possible, measured diameter at breast height (1.37 m) to 1 cm accuracy, total height to the nearest meter with a Relaskop, recorded decay class [48], and the number and types of defects (e.g., conks, forks, broken tops, cavities). Ground cover within the 16-m fixed plot included shrubs, grasses, and forbs. We defined shrubs as woody vegetation <4 m in height. For each species, we estimated cover to the nearest 5%. We classified downed wood (>10 cm) to species (if possible) and decay class [48], we measured length to the nearest meter, and measured diameter at both the small and large ends to the nearest cm. We estimated basal area for live trees and snags using a 40-factor prism and measured the diameter of trees within each plot to the nearest 0.25 cm. We also recorded species of live trees and snags, crown dominance [49], presence of defects (e.g., conks, forks, cavities), canopy base and tree height using a Relaskop to the nearest meter [50]. We did not collect canopy cover data. Instead, we joined our point locations with estimates from publicly available Gradient Nearest Neighbor data updated in 2012 [51]. We also separately report canopy cover estimates at marten locations in the Central Coast study area using publicly available LiDAR data [52].

We quantified vegetation plot data in two ways because the design differed between Humboldt marten locations (a point) and owl home ranges (an area). The spotted owl study was nearly complete when marten surveys began. Vegetation plots in the spotted owl study area were completed on a systematic grid with 150 m spacing starting at a random point throughout each spotted owl’s home range (n = 2,987 plots). We restricted vegetation plots to include only locations within each spotted owl’s 50% core area because these areas often include unique structures used for nesting, roosting, and provisioning young [16, 53, 54]. As the individual Humboldt marten was unknown for the scent detection survey locations, we used a new design to collect vegetation data at each marten location. Using the previously collected spotted owl data, we assumed that 4 stations would be needed to approximate vegetative conditions within a used stand (e.g., basal area, shrub cover). As such, at each Humboldt marten location we collected plot data at the point location and 100 m in 3 directions–to the north (0°), southeast (120°), and southwest (240°).

Because vegetation data were generally non-normally distributed and we expected a range of vegetative conditions within each study area, we calculated the median and interquartile ranges for each covariate by study area (Table 1). For the Humboldt marten data, we averaged each covariate at 4 vegetation locations at each site and described conditions between sites. For the spotted owl data, we averaged all locations within each core area and described the vegetation covariates between individuals. We displayed the distribution of our plot data. For metrics in which local models were available, we graphically included values for predicted suitable and highly suitable northern spotted owl habitat [7].

**Table 1:** Forest structure covariates used with MaxEnt modeling software v3.3 [55] to estimate probability of presence within modeling regions occupied by both Humboldt martens and spotted owls. Data from Davis, Hollen, [7] using Gradient Nearest Neighbor vegetation data, incorporating remotely sensed Landsat imagery and ground-based plot (Forest Inventory Analysis) updated in 2012 [51].

Covariate	Description
Canopy cover of all conifers	Percentage of conifer cover in the canopy as calculated using methods in the cover of Forest Vegetation Simulator
Diameter diversity index	A measure of the structural diversity of a forest stand based on tree densities in different diameter at breast height (~1.37m) classes. Calculation procedures are described in appendix 1 of McComb et al. (2002).
Stand height	Mean height of dominant and codominant trees
Conifer diameter	Mean conifer basal area weighted mean diameter at breast height of all live conifers
Large tree density	Estimated tree density for all live conifers ≥ 51 cm diameter at breast height.

Our goal in comparing vegetation characteristics across study areas was to evaluate the degree to which used Humboldt marten areas were contained within the range of predicted habitat suitability for spotted owls and whether those patterns were consistent across regions. Davis, Hollen, [7] summarized habitat analyses created from spotted owl location data collected during long-term demographic studies paired with remotely sensed data (1994–2012), providing estimates of the relative suitability (e.g., unsuitable, suitable, highly suitable) by geographic region (e.g., [7], Table 3.1, page 39). Predicted habitat suitability for spotted owls reported by Davis, Hollen, [7] has been applied in other analyses (e.g., [17]) and is publicly available. Because the Davis, Hollen, [7] model used remotely sensed data, we assumed comparison of vegetation plots at the nearby spotted owl study area would be useful as these data represent the largest number of vegetation plots in areas used by either northern spotted owls or Humboldt martens.

We included vegetation characteristics that could be manipulated by managers. We quantified vegetation metrics associated with the overstory (i.e., trees and snags), which are focal to describing spotted owl habitat [7], and understory (i.e., shrub and log characteristics) as such complexity has been associated with Humboldt martens in California [56]. We display overstory conifer features (i.e., canopy cover, basal area, diameter diversity, height, size) because these features are used in routine stand exams to plan timber sales and guide development of silvicultural prescriptions for mechanical thinning. Snags are a focus of retention and creation in federal timber sale planning due to their importance for a large variety of wildlife for nesting/denning and foraging [57, 58]. Post-treatment densities of snags, canopy cover, basal area, diameter diversity, height, and size are necessary information for U.S Fish and Wildlife Service Endangered Species Act Section 7 consultations for projects affecting spotted owl habitat. We used two size classes to count the number of large (>51 cm diameter) and very large (>76 cm) trees, snags, and logs per hectare to compare with Davis, Hollen, [7, 25] and state Forest Practices guidelines [59].

### Comparing predicted Humboldt marten and spotted owl occurrence with established late-successional reserves

We used MaxEnt modeling software v3.3 [55] to estimate the probability of presence within modeling regions occupied by both Humboldt martens and spotted owls [60]. For both species, we fit models using linear, product, and quadratic response functions and the same modeling parameters (e.g., regularization multiplier setting) similar to techniques used within our baseline spotted owl information [7]. We produced species distribution maps from all models using the maximum training sensitivity plus specificity threshold, which minimizes both false negatives and false positives [55]. We evaluated the area under the receiver operating curve (AUC) statistic to determine model accuracy and fit to the testing data [61]. The AUC statistic is a measure of the model’s accuracy, producing an index value from 0.5 to 1 with values close to 0.5 indicating poor discrimination and a value of 1 indicating perfect predictions [62]. We then estimated, for each region, the proportion of predicted occurrence area for Humboldt marten within established late-successional reserves. To test our method, we also confirmed whether the proportion of predicted spotted owl occurrence areas were inside these same established late-successional reserves.

We used a portion of the Humboldt marten location data as response variables to train, then the rest of the data to test a species distribution model for both areas using a suite of spatially referenced data layers (Table 1). We delineated modeling regions with a 10-km buffer around all Humboldt marten locations within each study area with the distance based on martens’ maximum daily distance movements [63]. Within these same geographic extents, we compiled spotted owl location data acquired from a series of public land and demographic surveys [7] to generate the species distribution model with the same suite of covariates (Table 1).

### Ethics statement

We obtained all necessary permits for this study, which complied with all relevant regulations. We captured and processed Humboldt martens in the Central Coast using methods described in Mortenson and Moriarty [32], which were approved by the USDA Forest Service’s Institute for Animal Care and Use Committee (USFS 2015–002) with an Oregon Department of Fish and Wildlife Scientific Take Permit (119–15). We captured spotted owls and attached radio-transmitters according to accepted procedures and practices for northern spotted owls (Forsman 1983; USGS Banding Permit no. 22514; USFWS Recovery Permit no. TE-99618A-4). We followed recommendations for the ethical use of wild animals for research [64–66].

## Results

We collected vegetation data within 537 vegetation plots within the 50% utilization distribution of 13 northern spotted owls home ranges (*x* = 41.3 plots/individual, range 11–79).

In the Central Coast we collected vegetation data at Humboldt marten resting locations with a known structure (n = 53 locations, average±SD = 4.8±2.2 per marten, n = 11 martens, S1 Table). We collected vegetation data at 16 additional locations where latrines were present or where at remote cameras that were visited repeatedly. Most vegetation data (77%) were in lodgepole dominated stands within the Oregon Dunes Recreation Area (41 sites, 165 plots) with the remaining 23% were in Sitka spruce or Douglas-fir dominated stands (12 sites, 47 plots), where some plots would be different types (S1–S3 Figs).

We collected vegetation data at 47 sites where Humboldt martens were detected with genetically verified scats (n = 34) and at remote camera locations (n = 13) in the South Coast study area (188 plots). Of these areas, 26% (11 sites, 41 plots) were in areas with serpentine soils (S1–S3 Figs).

### Fine-scale vegetation characteristics

**Fig 2:**
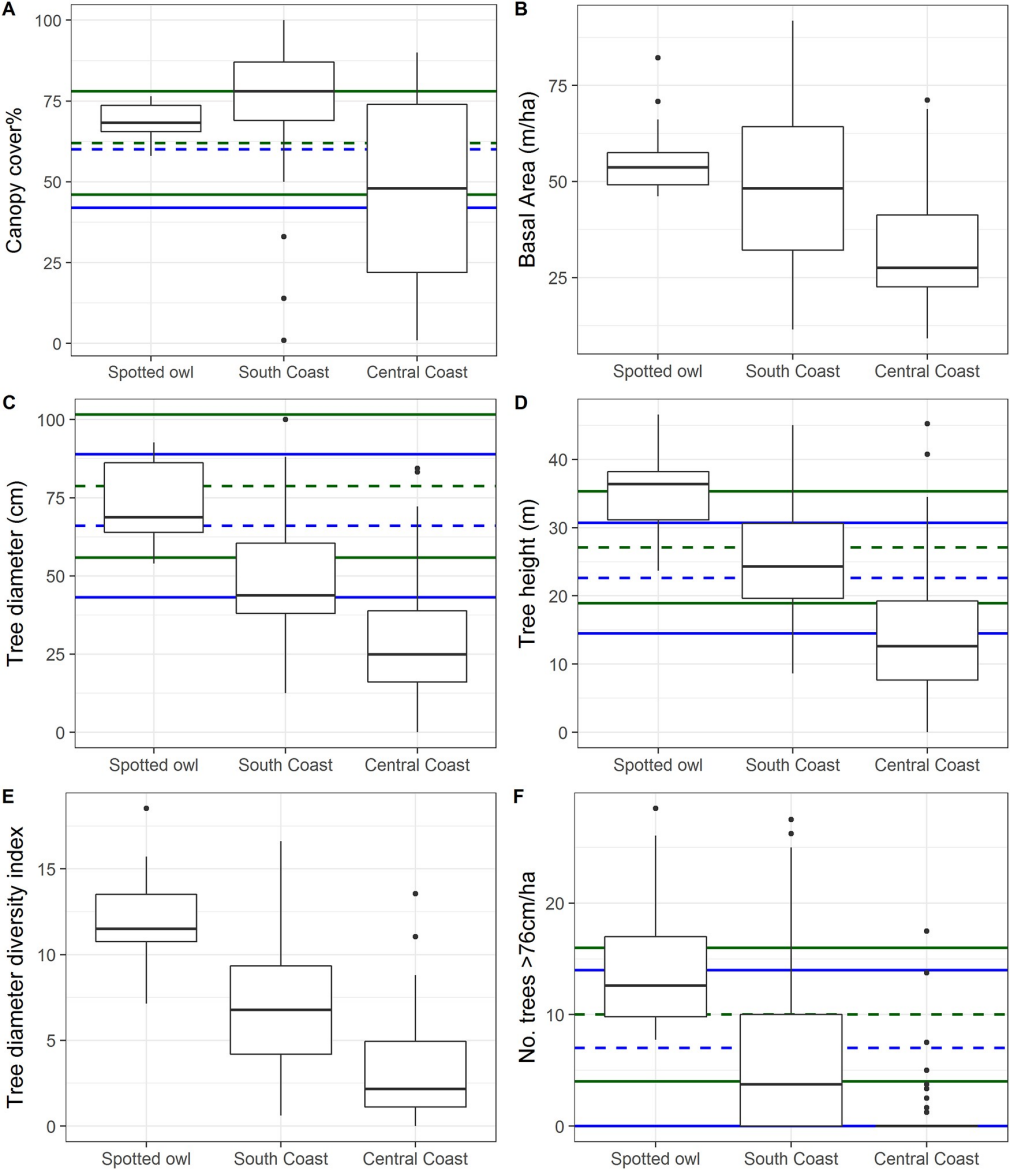
Vegetation data—Tree and canopy. Comparison of tree and canopy metrics at northern spotted owls (*Strix occidentalis caurina*) and a coastal subspecies of Pacific marten (*Martes caurina humboldtensis*) sites in coastal Oregon. Box plots show maximum and minimum values (end of lines or extreme points), first and third quartiles (top and bottom line of box), and the median (line within box). In some cases (A, C, D, F), a variable was also in a top model describing predicted habitat [7]. We show the mean (dashed line) and 95% confidence interval (colored line) for predicted high (blue) and very high (green) quality spotted owl habitat.

**Fig 3:**
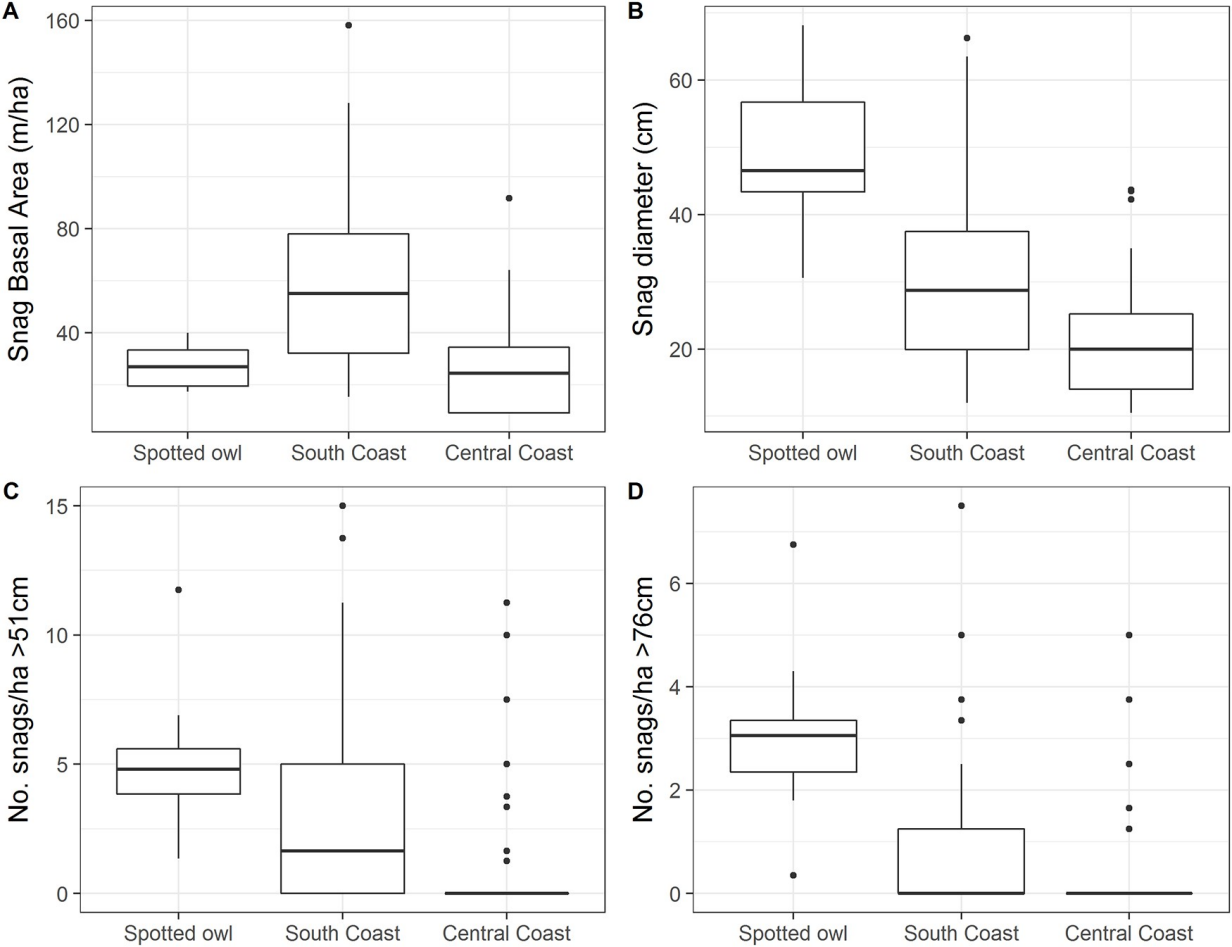
Vegetation data—Snag. Comparison of snag metrics at northern spotted owls’ (*Strix occidentalis caurina*) and a coastal subspecies of Pacific marten (*Martes caurina humboldtensis*) sites in coastal Oregon. Box plots show maximum and minimum values (end of lines or extreme points), first and third quartiles (top and bottom line of box), and the median (line within box).

**Fig 4:**
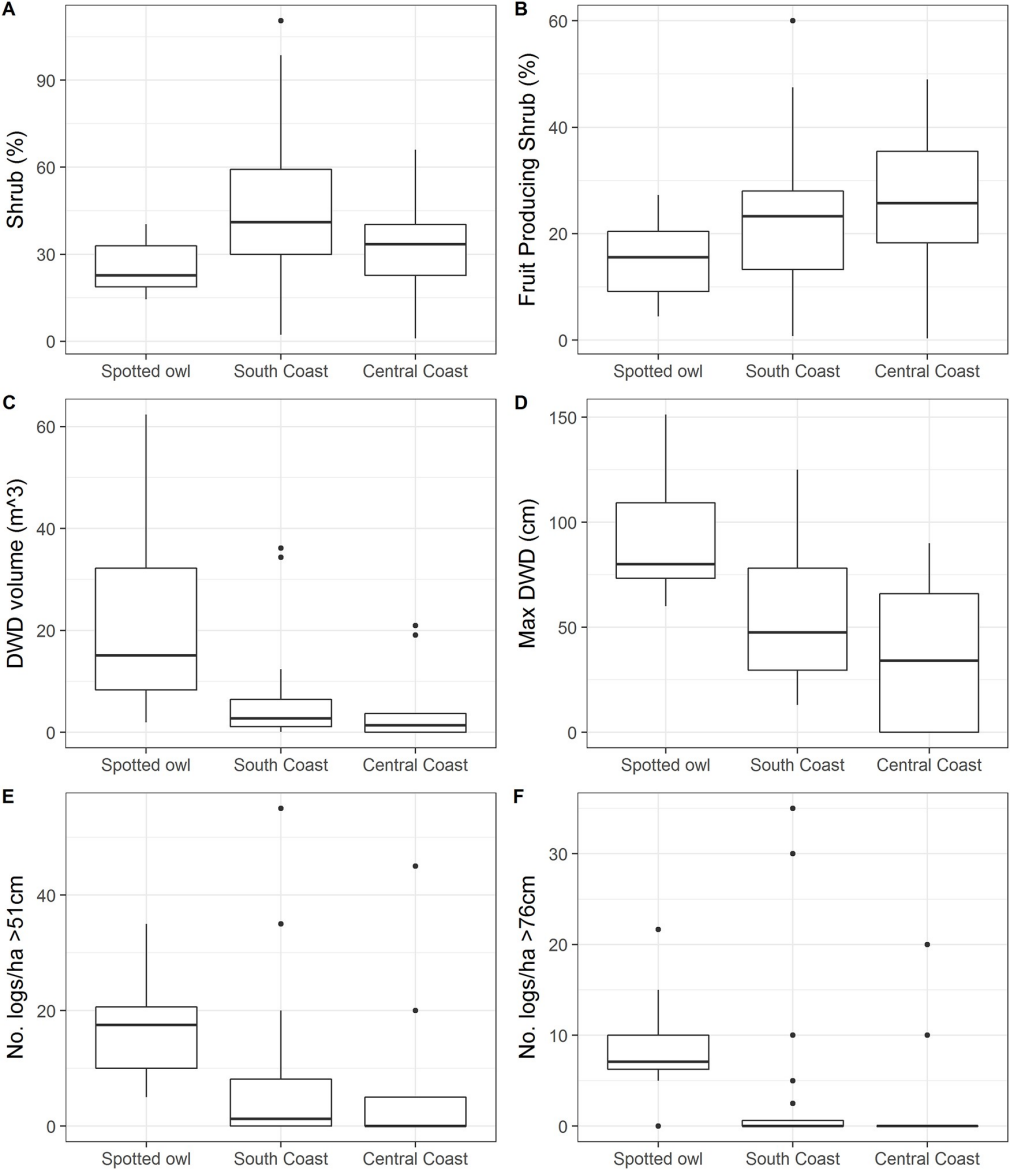
Vegetation data—Understory. Comparison of understory shrubs and logs, including downed woody debris (DWD) characteristics at northern spotted owl (*Strix occidentalis caurina*) and a coastal subspecies of Pacific marten (*Martes caurina humboldtensis*) sites in coastal Oregon. Box plots show maximum and minimum values (end of lines or extreme points), first and third quartiles (top and bottom line of box), and the median (line within box).

Overall, we found a trend of higher decadence (e.g., larger trees, snags, and logs, taller trees, increased diversity) and decreased variation in vegetative conditions (i.e., range of values) used by spotted owls as compared to Humboldt martens (Figs 2–4). For instance, canopy cover was high for spotted owl use sites (quartile range 65–73%) in the South Coast Humboldt marten study area (range = 69–87%) but generally lower and more variable in the Central Coast Humboldt marten study area (range = 22–74%; Fig 2a). Basal area varied broadly in the South Coast study area, a result suggesting substantial variation among marten sites. Median canopy cover, tree diameter, tree height, and number of large trees from the South Coast Humboldt martens were within the range of predicted suitable and highly suitable spotted owl habitat (Fig 2). Our data showed a similar relationship as we found spotted owls in areas with the largest and tallest trees (Fig 2c and 2f). The number of very large trees, snags, and logs (>76 cm) per acre were highest in spotted owls’ core areas, lower at sites with Humboldt martens in the south, and lowest in the Central Coast (Figs 2f, 3d and 4f).

Snag characteristics, such as diameter, varied substantially between study areas (Fig 3b–3d) but basal area overlapped across study areas (Fig 3a). Most snags were small in diameter (Fig 3b) but within the same sites as several large (>51 cm; range = 3.9–5.6, 0–5, 0 range of structures/ha for spotted owl, South and Central Humboldt marten study areas) and very large (>76 cm; range = 2.4–3.4, 0–1.3, 0 structures/acre for spotted owl, South and Central Humboldt marten study areas) snags.

**Fig 5:**
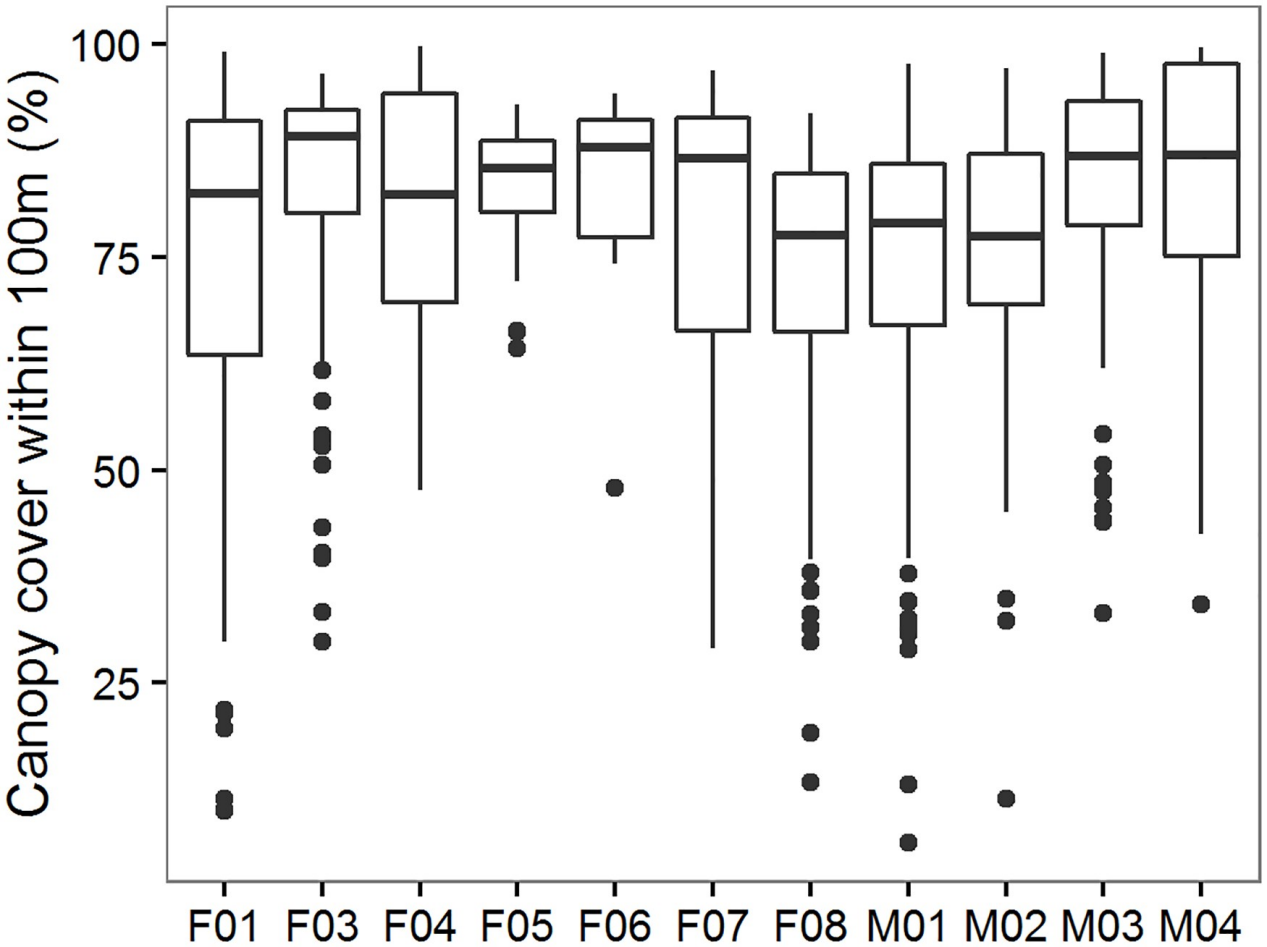
Vegetation data—Humboldt marten fine-scale canopy association. Percent canopy cover (vegetation height >1 m estimated from LiDAR) within a 100 m moving radius of spatial locations of 11 Humboldt martens (*Martes caurina humboldtensis*, x-axis, F = female, M = male) in the Central Coast, Oregon. Data were collected using VHF and GPS telemetry from October 2015 to April 2016 (see methods within [67]). Here, the combination of both overstory and understory cover used by Humboldt martens was high (average median = 81%, range = 64–92%). Box plots show maximum and minimum values (end of lines or extreme points), first and third quartiles (top and bottom line of box), and the median (line within box).

Understory composition included both shrub cover and logs. Shrub cover quartiles ranged from 19–59%, with the South Coast Humboldt marten population having the most consistently dense shrub cover (Fig 4a). Fruit-bearing shrub cover was highest in the Central Coast (Fig 4b, Quartile Range, Q_r_ = 18–35%) and was largely represented by shade-tolerant species (e.g., salal). Humboldt martens’ telemetry locations in the Central Coast study area were strongly associated dense vegetation >4m high, which included both shrub and overstory canopy as estimated from aerial LiDAR (Fig 5). Log volume, size, and number (Fig 4c–4f) were similar in trend to tree and snag metrics with the highest quantities and sizes in the spotted owl study area followed by south and Central Coast Humboldt marten areas. More logs and larger logs were present compared to either live trees or snags (>51 cm; Q_r_ = 10–26, 0–8, 0–5; >76 cm; Q_r_ = 6.3–10, 0–0.6, 0 for the spotted owl, South and Central Humboldt marten study areas), suggesting a prior legacy of larger structures that are no longer being recruited in contemporary stands.

### Comparing predicted Humboldt marten and spotted owl distribution with established reserves

**Fig 6:**
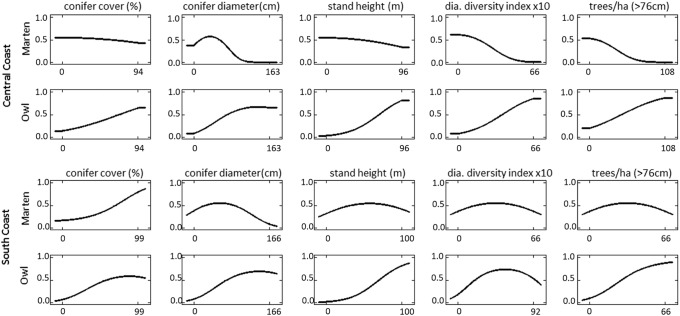
Humboldt marten and spotted owl distribution predictor response. Individual predictor variables response functions for each species within two modeling regions (south and Central Coast). Predictor variables for northern spotted owls (*Strix occidentalis caurina*, Owl) and a coastal subspecies of Pacific marten (*Martes caurina humboldtensis*, Marten) were generated from remotely sensed data [7] and included percent canopy cover, diameter at breast height, average tree height (stand height), diameter Diversity Index (dia. Diversity index) and trees per hectare (ha). Additional descriptions of variables are found in Table 1.

**Fig 7:**
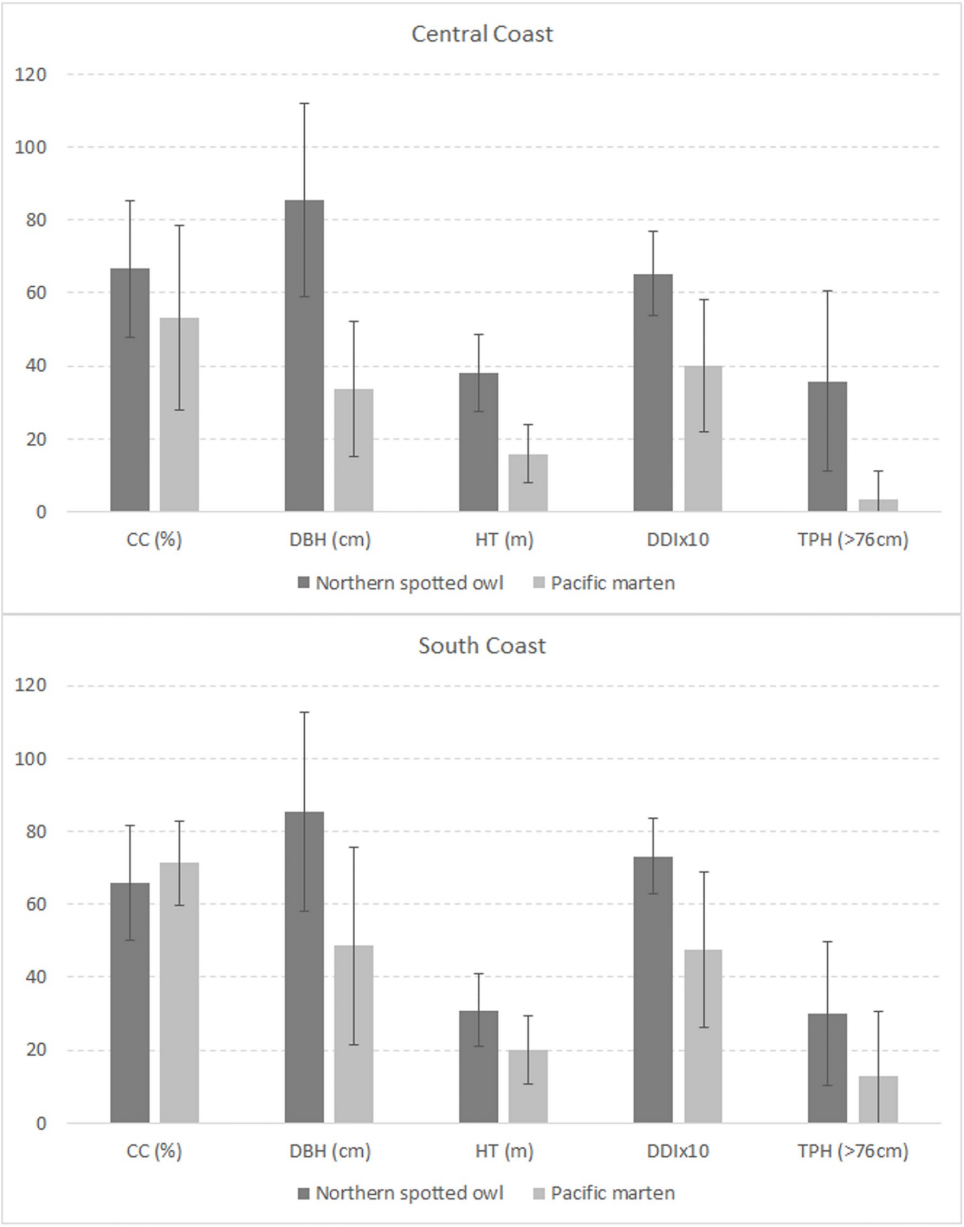
Humboldt marten and spotted owl distribution prediction averages. Mean (±1SD) for each covariate used in predicted use areas for northern spotted owls (*Strix occidentalis caurina*) and a coastal subspecies of Pacific martens (*Martes caurina humboldtensis*). Variables were selected from remotely sensed data [7] and included canopy cover (CC), diameter at breast height (DBH), tree height (HT), Diameter Diversity Index (DDI) and Trees Per Hectare (TPH) with descriptions of variables located in Table 1.

**Fig 8:**
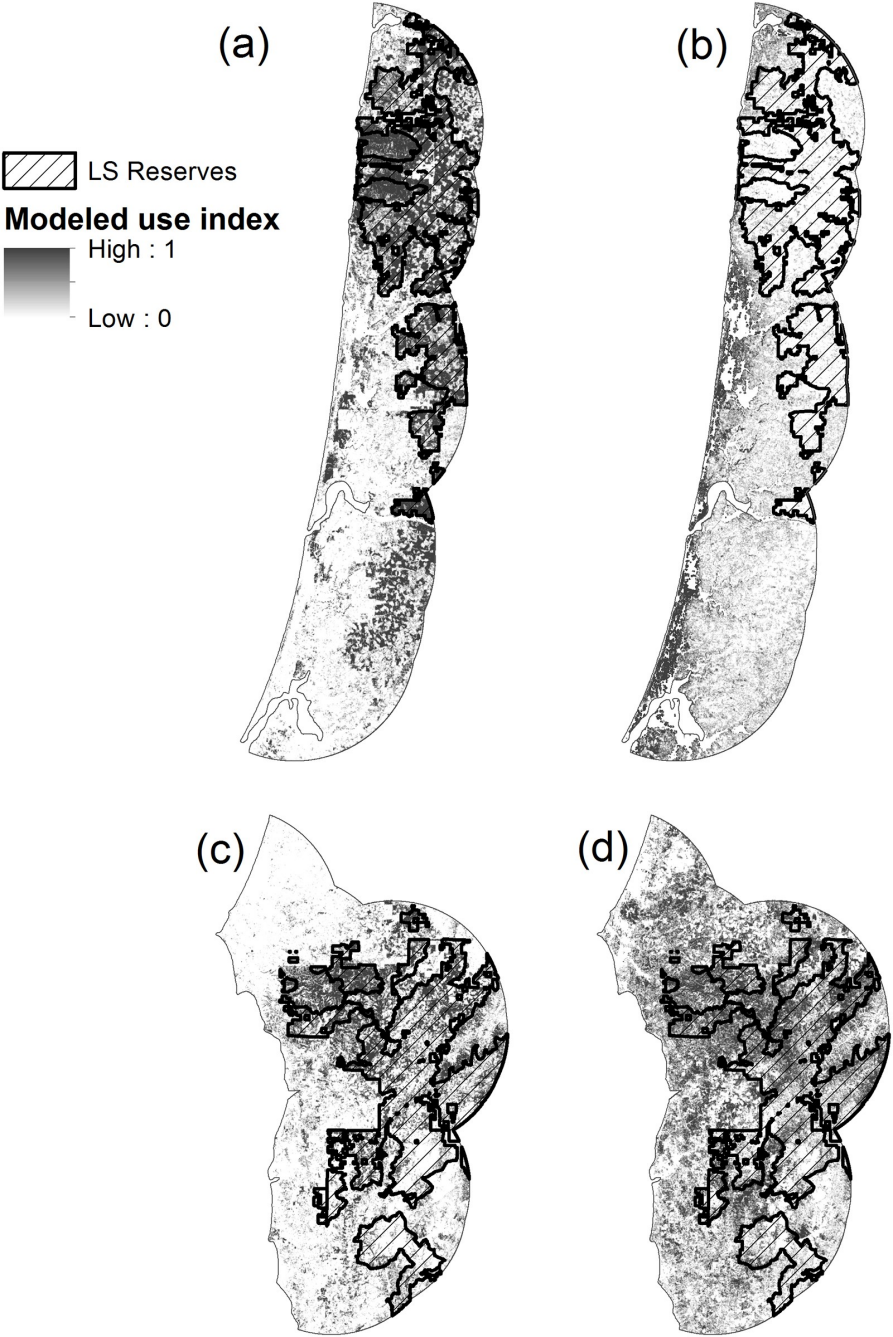
Humboldt marten and spotted owl distribution models. Species distribution models based on observed species use vs. available for use. Top maps show relative likelihood of use for northern spotted owls (a) and a coastal subspecies of Pacific martens (b) in the Central Coast. Bottoms maps show relative likelihood of use for northern spotted owls (c) and Humboldt marten (d) in the South Coast. Cross-hatched areas were designated as late-successional forest reserves under the 1994 Northwest Forest Plan.

All models fit the training data well with AUC values ranging from 0.76 to 0.84 (Table 2). Spotted owl distribution models were more similar between modeling regions whereas predicted Humboldt marten distribution models were similar to spotted owls in the South Coast but not in the Central Coast (Figs 6–8). Broad scale predictor variable values for each species (Fig 6) overlapped with our observed fine scale “tree/overstory” metric (e.g., Figs 2a and 7). Humboldt martens in the South Coast appeared to have thresholds within their predicted habitat such that stands were often neither young nor old, but reached the highest predicted value at moderate diameters, heights, diversity indices, and expected numbers of large trees (Fig 6a). Similar to trends observed in the vegetation plot data, Humboldt martens in the South Coast were predicted to be in areas with smaller tree sizes, shorter tree heights, and fewer large trees (Fig 6c). However, shrub cover was unavailable with GNN remotely sensed data (see Fig 5, [51]). Predicted spotted owl distribution modelled similarly in both the Central and South Coast study areas [7]. The overall trends observed with both fine-scale vegetation (Fig 2) and this analysis (Figs 6 and 7) were similar for both species.

**Table 2:** Maxent modeling statistics for northern spotted owls (*Strix occidentalis caurina*) and coastal Pacific marten (*Martes caurina humboldtensis*) in the Oregon coast range. The area considered was within 10km of known marten locations, using spotted owl data from demographic studies [7].

	Training locations	Area under the Curve
South Coast		
Humboldt marten	46	0.760
Spotted owl	35	0.839
Central Coast		
Humboldt marten	53	0.831
Spotted owl	25	0.835

Predicted Humboldt marten distribution was not captured by the reserve design of the Northwest Forest Plan but, as expected, predicted spotted owl use was fairly well represented within reserves. In the Central and South Coast study areas, predicted distribution (>60% threshold) for spotted owls was 47% and 49% within federal reserves (36,697 and 41,316 ha, respectively). In contrast, predicted Humboldt marten distribution differed greatly from those for northern spotted owls in both the Central Coast (13% overlap in 4,747 ha) and the South Coast (34% overlap in 20,867 ha) (Fig 8).

## Discussion

Conservation efforts for single species largely influence forest management decisions on public lands in the Pacific Northwest, USA (e.g., [25, 68]). Our analyses indicated that two late successional associated species were not predicted to use the same areas. Sites used by both northern spotted owl and Humboldt marten had overlapping vegetation characteristics, such as overstory cover, but the species were associated with differing sizes and amounts of large trees, snags, and logs. Thus, areas used by spotted owls for nesting and roosting represented only a portion of the broader vegetation conditions used by Humboldt martens. In contrast to areas used by owls, Humboldt martens in both the South Coast and Central Coast regions used areas with dense and diverse shrub communities. Shrub cover may be a surrogate for the structural complexity typically provided by downed logs, both of which provide protective cover and foraging opportunities for the small-bodied terrestrial carnivore [69]. Humboldt martens may also use areas predicted for spotted owls and older forests, but we did not observe such overlap in coastal Oregon. Our study locations may be more diverse and have a different management history compared to locations where Humboldt martens were found in northern California [56, 70]. If critical habitat elements were not strongly correlated with vegetation cover, but instead associated with more ephemeral resources such as prey [71] and predation risk [72], then we would predict Humboldt martens to be associated in areas with such biotic elements as much or more than areas with vegetation cover [73]. Understanding the proportional importance of individual habitat elements was beyond the scope of this study, but experimental studies elucidating such information would benefit future management and conservation (e.g., [4]).

During our study, spotted owls used areas with dense canopy cover, large tree diameters, and tall trees as predicted by Davis, Hollen, [7]. These results align with results from 4 demographic studies that showed spotted owls were most associated with older and taller trees [74, 75]. Such tall, robust structures likely provide thermal gradients and microclimates suitable for owls that vary within the canopy (e.g., [76]); such variation may be especially important in warmer climates or southern latitudes. Our study included both fine-scale vegetation descriptions and regional-scale distribution models, which we used to describe the range of conditions used by Humboldt martens and northern spotted owls. Results at both scales were similar (Figs 2 and 6) and indicated that owls were consistently using older forests and a narrower range of conditions than martens (e.g., [7]).

Relative to the umbrella species concept, our data reveal the importance of understanding detailed life history requirements, and how a species exploits available resources, before assuming habitat conservation for a second species will benefit both species (see also [77–79]). Currently, management for the northern spotted owl focuses on silviculture treatments to create or maintain “nesting-roosting” stands, characterized as conifer stands with a multi-layered, multispecies canopy dominated by large (>76.2 cm diameter) conifer overstory trees, a shade-tolerant understory, substantial decadence, large accumulations of logs and other woody debris, and a canopy that is open enough to allow northern spotted owl flight patterns [80]. Although Humboldt martens may use these stands, our analyses suggest that Humboldt martens exist in areas with a higher diversity of conifer size classes as long as expansive dense shrub cover, predominantly in the form of tall and contiguous salal and evergreen huckleberry, are also available. In these areas, ground-level prey may be unavailable to foraging spotted owls.

Both spotted owls and Humboldt martens used areas containing large snags and logs. We found both species in areas with >1 large snag and log (>51 cm) and often in areas with >1 very large snag and log (>76 cm). These results were surprising, given that Humboldt marten populations occupy areas with poor soils and presumably slower tree growth rates compared to the spotted owl study area. Both spotted owls and Humboldt martens show strong affinities to large, tall trees for nesting, denning, and resting [70, 74]. The presence of large live trees may support recruitment of snags and logs. During this study, most Humboldt marten sites were located on public lands. Maintaining 3–6 large live trees (>76 cm) may be a challenge on adjacent more intensively managed ownerships which minimally serve as corridors between larger areas of predicted habitat. Oregon Forest Practice Rules require 5 green trees and snags per hectare >28 cm and 2 logs each with a volume of ~ 10 cubic feet, or as small as 20cm in diameter [59]. In contrast, our data suggested that used sites had a median of 17 and 3 logs >51 cm in diameter per hectare for spotted owls and Humboldt martens respectively, amounts exceeding the size classes required in Oregon.

We used presence-only modeling to evaluate the amount of predicted suitable area within reserve systems for each species. The reserves established under the Northwest Forest Plan (1994) provided a sizable amount of area predicted for spotted owl occurrence but not for Humboldt marten–especially in the Central Coast region. The central Humboldt marten study area in the Oregon Dunes contained young (<70 years) and sparse trees [30]. The study area’s vegetation characteristics contrasted with older forest characteristics in areas used by spotted owls and Humboldt martens in the South Coast population. This difference represents an opportunity to quantify variation in habitat conditions and highlight the importance of including heterogeneity in planning for treatments in managed forest landscapes. For instance, canopy cover was moderate to low in the central and South Coast Humboldt marten sites (Fig 2a), but when cover estimates included both shrubs and over story trees, Humboldt martens used areas with >75% cover (e.g., Fig 5) which was expected for their habitat needs [81, 82]. We suggest that simplifying vegetation characteristics into classes or broad categories would be insufficient for describing conditions where northern spotted owls and Humboldt marten can persist. Overstory vegetation conditions may mask the importance of subdominant overhead cover that small mammals (10-1000g) likely use to avoid predation [83]. Berry or mast producing plants are correlated with increased prey for fishers [84, 85]. Additional food resources from berries and mast may support both high fisher [86] and marten densities [34] and increase opportunities for other carnivores [73, 87]. We suspect martens in the Central Coast have small home ranges in this atypical vegetation because of both dense shrub cover (reducing predation risk) and high availability of prey [34, 88]. This population is unique in North America, persisting in an area lacking both winter snow cover and often without late-successional conditions [81]. We suspect that spotted owls may not exhibit such predation- and competition-driven habitat plasticity [89], except in rare circumstances (e.g., [16, 90]), but could benefit from increased heterogeneity when it increases small mammal densities. As such, a combination of vegetation at each strata (overstory, mid-story, understory) and biotic information (prey availability) should increase the accuracy of species occurrence predictions, and better inform management goals for population distribution and abundance.

Despite perceived similarity in structural elements associated with spotted owls and Humboldt martens, we demonstrated that Humboldt martens in Oregon used a much broader range of vegetation types than spotted owls. Our Humboldt marten use locations diverge from conditions described in northern California where Humboldt martens were associated with serpentine soils and old forested stands [56, 70] as our Humboldt marten locations were outside the Siskiyou serpentine ecoregion with few sites in similar soil types. We acknowledge limitations to this study, largely our relatively small sample size and lack of paired random locations for our vegetation plots. Although we completed vegetation sampling at a census of all known Humboldt marten locations, quantification of marten vegetation use in the future may best be represented by adult animals with known activities (e.g., denning). Still, our results represent a broad spectrum of conditions used by Humboldt martens for foraging, assumed kit rearing, and resting. Secondly, we were unable to perform selection analyses at the plot scale for vegetation characteristics. This was purposeful, largely due to logistic constraints and limited resources. Because our vegetation data were collected using strict protocols, our study could be replicated or built upon for new objectives. As such, we expect this study provides value for managers, and we believe the collective scientific community will be able to build from our work.

## Conclusions

We can use our descriptions of vegetation structure to identify forest types and ownerships where heterogeneity can be achieved; broadly classified in three areas for these species: areas with Humboldt martens but not spotted owls (e.g., the Central Coast Humboldt marten study area), areas with Humboldt martens and spotted owls (e.g., the South Coast Humboldt marten study area), and areas with spotted owls but not Humboldt martens (e.g., the Coos Bay spotted owl study area).

We found Humboldt martens using young forests with interconnected dense, patches of shrubs. We propose that such conditions likely limit aerial predation for raptors and would not be considered suitable for spotted owls. Reducing dense cover and connectivity, especially ground-based vegetation such as shrubs and downed logs increases vulnerability of Humboldt martens, likely similar to martens in mountainous environments (e.g., [91]). Martens in higher elevation, snow-dominated regions appear sensitive to reductions in canopy cover; a 25% decrease in canopy cover has been correlated with population declines [92–94]. However, these coastal Humboldt martens may be able to use areas with contiguous shrub cover and lower canopy cover. Thus, we propose that harvest practices that less dramatically alter the overstory while encouraging dense shrub growth, particularly salal and evergreen huckleberry [95], and retain or increase large downed woody material [96, 97] would provide benefits to coastal Humboldt marten populations. Nonetheless, we caution that our observations in the Central Coast were often in areas with coastal fog and significant rainfall coupled with sandy soils that may provide a unique growing environment conducive to tall, dense, shrubs which may not grow well elsewhere.

In areas with or near both Humboldt martens and spotted owls, such as in the South Coast Humboldt marten study area, a combination of strategies could be considered. Our spotted owl plots were within areas with many large and tall trees. Retaining and recruiting large structures would benefit both Humboldt martens and spotted owls (e.g., [70, 74]). Similarly, both Humboldt martens and spotted owls occupied areas with numerous large (>76 cm) logs. Increasing heterogeneity in sympatric areas by encouraging areas of dense, fruit-producing shrubs and small openings may provide habitat conditions for small mammals. North, Kane [74] observed spotted owls predominantly in areas with closed canopies, but with openings <1000m^2^. Optimal levels of vegetative heterogeneity and foraging habitat have not been evaluated for either Humboldt martens or spotted owls. However, based upon management recommendations in the Northern Spotted Owl Recovery Plan [98], creating of patches of early seral forest outside of core home ranges (e.g., 800m radius around nest site) is an acceptable management option, which would diversify spotted owl habitat conditions and increase heterogeneity and shrub cover for Humboldt martens.

Federal lands that only support spotted owls and are beyond the dispersal distance for Humboldt martens are regulated under the Northwest Forest Plan [9], and we observed alignment with reserves and predicted habitat quality. Northern spotted owls in our study used a narrow band of vegetation characteristics and may be more restricted than Humboldt martens in their dependence on old forests.

Both species were associated with forest cover from trees, shrubs, or both strata. In Oregon, for example, restoration and management would benefit from strategies for each ownership type as ownership largely determines the age, size, and distribution of forests condition. For instance, lands with the primary goal of wood production could consider retaining the same structures over multiple harvests, especially in areas adjacent to Humboldt marten and spotted owl populations. Depending on patterns of land ownership and management intensity, landscape scale heterogeneity may be accounted for by nature of ownership (e.g., BLM land surrounded by private lands). Here, increasing shrub cover or windrows [97, 99] and connectivity for Humboldt martens could be beneficial both within patches and within a potential home range. Conversely, the Forest Service manages more contiguous forests (i.e., not in a checkerboard configuration like much of BLM lands) that contain extensive blocks designated as Late-Successional Reserves. Restoration in some areas could include increasing heterogeneity in the form of openings within a stand (<1000m^2^) representing less than 25% of a marten home range, outside of the core use areas of known spotted owl sites.

## Supporting information

S1 Fig Vegetation data—Tree and canopy boxplots with additional stand divisions. (DOCX)

S2 Fig Vegetation data—Snag boxplots with additional stand divisions. (DOCX)

S3 Fig Vegetation data—Understory log and shrub boxplots with additional stand divisions. (DOCX)

S1 Table Summary of marten resting location data in the Central Coast. (DOCX)

S1 Dataset Marten vegetation data and code. (ZIP)

S1 File Vegetation protocol. (PDF)
