# Supplementary material for: Describing vegetation characteristics used by two rare forest-dwelling species: Will established reserves provide for coastal marten in Oregon?
Source: PLoS One. 2019 Jan 31;14(1):e0210865. doi: 10.1371/journal.pone.0210865 (PMC6354973; doi:10.1371/journal.pone.0210865)

**S3 Figure. Vegetation data – understory log and shrub boxplots with additional stand divisions.** We compare site level (elevation) and tree and canopy metrics at northern spotted owls (*Strix occidentalis caurina*) and a coastal subspecies of Pacific marten (*Martes caurina humboldtensis*) sites in coastal Oregon. Box plots show maximum and minimum values (end of lines or extreme points), first and third quartiles (top and bottom line of box), and the median (line within box). We divided owl sites into stands dominated with Douglas fir (Spotted owl, DougFir; *Pseudotsuga men*ziesii, n = 13 individuals, 513 plots) or mixed conifer (Spotted owl, mixed, n= 11 individuals n = 24 plots). We separated marten sites in the South Coast in areas designated as “Serpentine soils” (Steve Campbell, Natural Resources Conservation Service Soils, created March 2018; Marten South, Serpentine, n = 11 sites, 41 plots) and Douglas fir dominated stands (Marten South, DougFir, n=47 sites, 188 plots). We separated sites within the Central Coast into stands dominated by either Sitka spruce (*Picea sitchensis*) or Douglas fir (Marten Central, SitkaDoug, n=12 sites, 47 plots) and shore pine (*Pinus contorta*, Marten Central, Pine; n = 41 sites, 165 plots). Due to the small sample size our inability to stratify by these classes intentionally, we view these characterizations as opportunities to visualize potential strata within each region, but we did not feel the data were sufficient for interpretation. We retain the mean (dashed line) and 95% confidence interval (colored line) for predicted high (blue) and very high (green) quality spotted owl habitat [2].


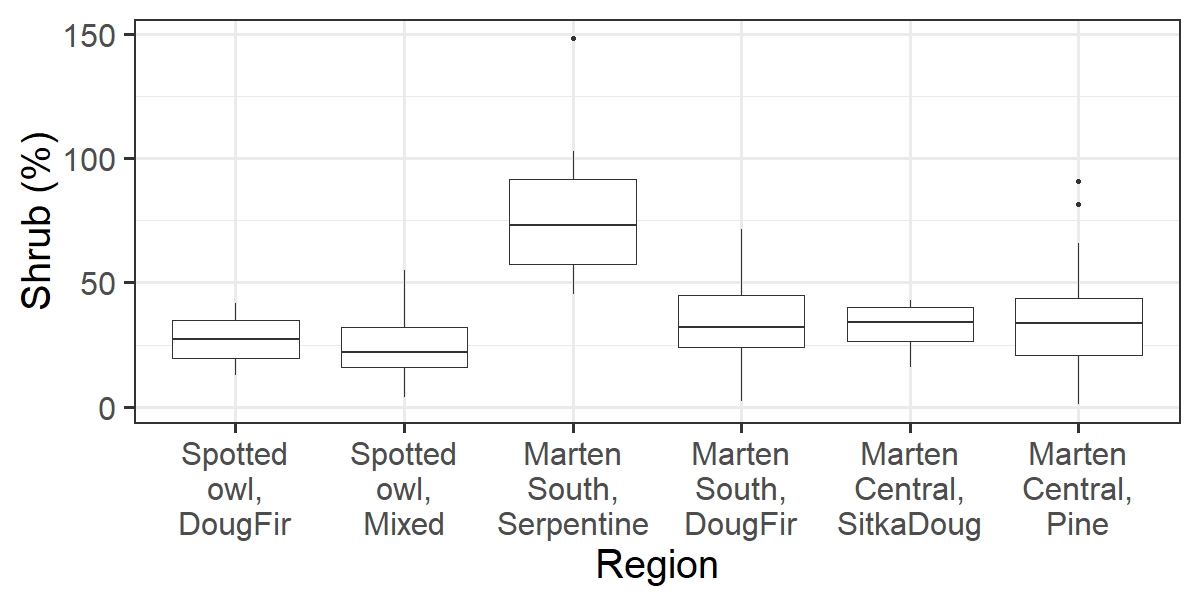


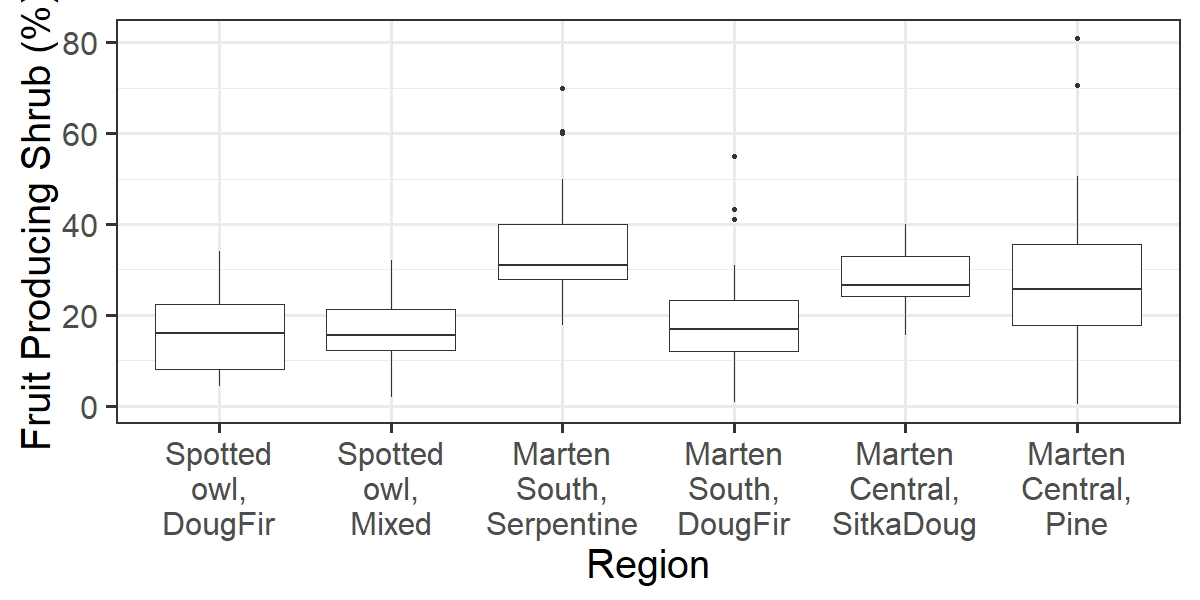


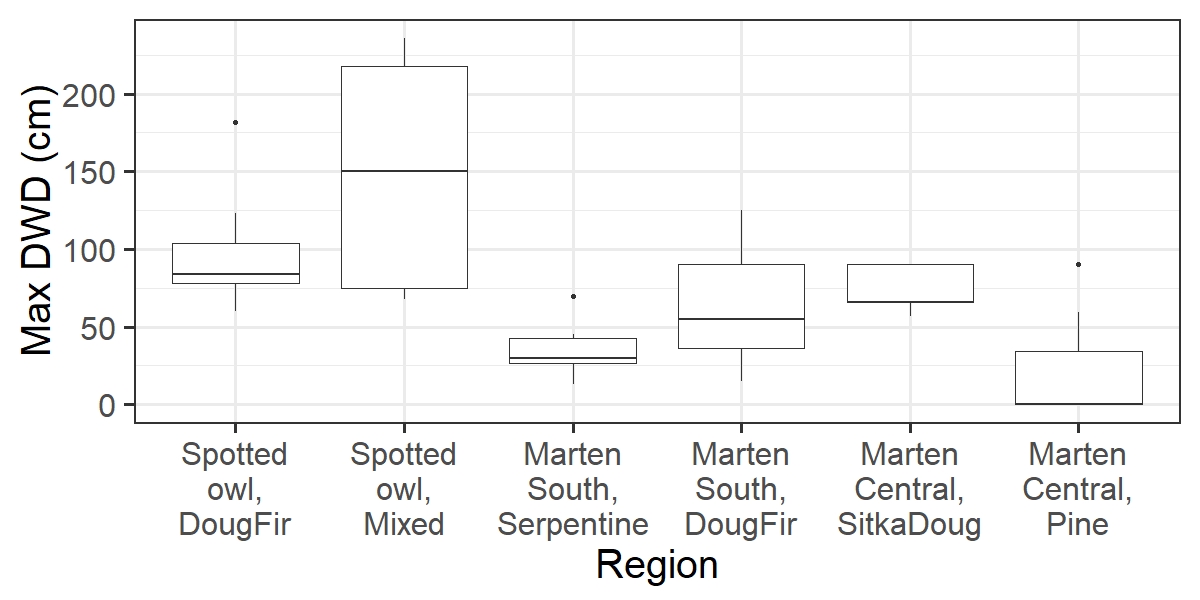

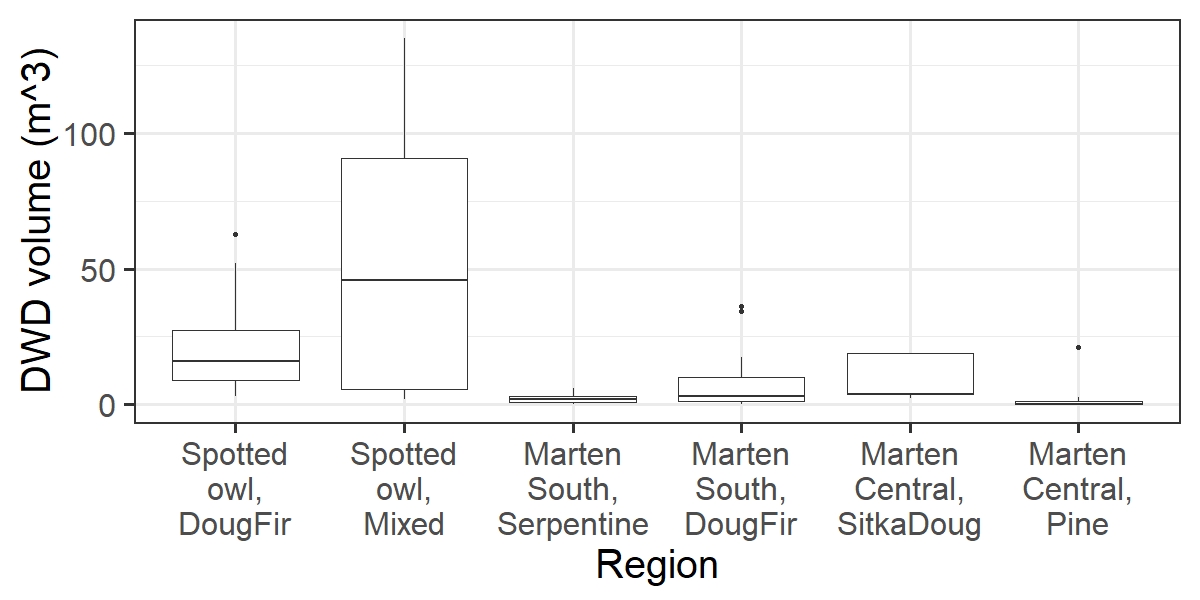

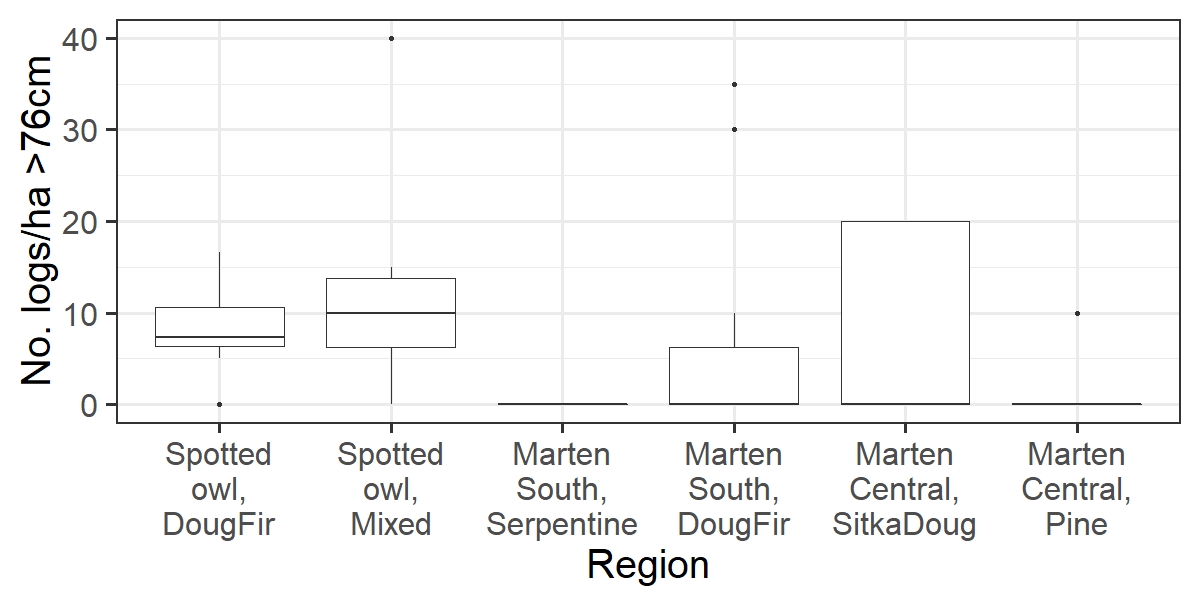

Supplement: S3 Fig — (DOCX) [file pone.0210865.s003.docx]
