# Supplementary material for: Describing vegetation characteristics used by two rare forest-dwelling species: Will established reserves provide for coastal marten in Oregon?
Source: PLoS One. 2019 Jan 31;14(1):e0210865. doi: 10.1371/journal.pone.0210865 (PMC6354973; doi:10.1371/journal.pone.0210865)
Supplement: S1 Table — (DOCX) [file pone.0210865.s004.docx]

**S1 Table. Summary of marten resting location data in the Central Coast.** We collected location information on Humboldt martens (*Martes caurina humboldtensis*), a subspecies of Pacific martens, within in the Oregon Dunes Recreation Area between October 2015 and April 2016 (see Linnell et al. 2018). We collected spatial locations (Locations) on marten when they were radio collared (Tracking period) with either a G10 snap technology GPS unit (27g, Advanced Telemetry Systems, “ATS”), a M1820 VHF unit made by ATS (27g; “VHF”), or a Quantum 4000 micro-mini GPS unit made by Telemetry Solutions (41-44g, “TS”). GPS data were collected for a short period of time (Tracking period) but individuals were monitored longer using VHF telemetry. We estimated territories using 99% Local Convex Hulls (LoCoH) and opportunistically found rest locations with an identified structure or the general vicinity (Number of rest structures or areas). We collected vegetation data only at known structures that were found before January 2016 (Vegetation sites).

| Marten ID | Tracking period | Locations | Collar Type | 99% LoCoh (km^2^) | Number rest structures or areas | Vegetation sites |
| --- | --- | --- | --- | --- | --- | --- |
| F01 | 11/22/15 - 01/05/16 | 2623 | ATS | 0.59 | 4 | 2 |
| F03 | 10/14/15 - 10/29/15 | 752 | TS | 0.62 | 10 | 7 |
| F04 | 10/14/15 - 11/25/15 | 37 | VHF | 0.79 | 7 | 5 |
| F05 | 10/18/15 - 01/19/16 | 33 | VHF | 0.71 | 4 | 4 |
| F06 | 10/29/15 - 12/29/15 | 23 | VHF | 0.27 | 7 | 4 |
| F07 | 10/29/15 - 01/27/15 | 35 | VHF | 0.84 | 13 | 9 |
| F08 | 11/29/15 - 12/24/15 | 2960 | ATS | 0.64 | 7 | 7 |
| M01 | 12/25/15 - 01/01/16 | 666 | ATS | 2.2 | 6 | 3 |
| M02 | 10/16/15 - 10/26/15 | 477 | TS | 2.2 | 6 | 6 |
| M03 | 11/30/15 - 12/05/15 | 324 | TS | 1.7 | 2 | 2 |
| M04 | 11/23/15 - 11/26/15 | 173 | TS | 1 | 4 | 4 |
| Total |  | 8103 |  |  | 70 | 53 |
| Average ± Standard Deviation | | 737±1051 |  | 1.1±0.7 | 6.4±3.1 | 4.8±2.2 |
