# Supplementary material for: Describing vegetation characteristics used by two rare forest-dwelling species: Will established reserves provide for coastal marten in Oregon?
Source: PLoS One. 2019 Jan 31;14(1):e0210865. doi: 10.1371/journal.pone.0210865 (PMC6354973; doi:10.1371/journal.pone.0210865)
Supplement: S1 File — (PDF) [file pone.0210865.s006.pdf]

S1 File. Vegetation Protocol used in PONE-D-18-17276

Spotted and Barred Owl Vegetation Survey Protocol with Marten sites added using the same vegetation data, but a differing distribution of plots (all known locations >240m were selected).

Data were collected 2014-2016

Contact: Drs. Jake Verschuyl or Katie Moriarty (jvschuyl@ncasi.org, kmoriarty@ncasi.org)

Distribution of Survey Plots

Habitat plots are distributed along a 120 meter grid within 95%MCP owl home ranges. Due to large size of home ranges in the Coos Bay study area it was not feasible to do all plots within the MCPs so some areas were cut.

Additional plot locations were provided by BLM. These were lidar ground truthing points within owl 95%MCP home ranges. These permanent plots were surveyed originally by BLM contractors in approximately 2010(?). Original plot centers were marked by white pvc pipe center stakes with pink flagging. Trees in the plot were marked by white spray painted numbers. Shrub information was not collected during the original plots. For the NCASI owl project, crews will relocate all lidar permanent plots within owl home ranges and complete a vegetation survey following the same protocol as for the 120m grid plots with the exception that plot centers will be placed exactly at the original center stake.

All plot data will be collected on paper data forms. Definitions/instructions for filling out the form are outlined below. Note: A fully trained observer should complete the General Plot Info, Trees, Snags, and Shrubs portion of each plot within 15 to 20 minutes. DWD data collection should take an additional 5 to 10 minutes.

General Plot Information

UTM: NAD83 UTM Zone10 coordinates of plot center.

Waypoints for plots are loaded into Garmin GPS units prior to field work. Navigate to each plot waypoint using Garmin. For the 120m grid plots, get within 10 meters or as close as safely possible to the waypoint. If the waypoint is on a cliff, then get as close as you can and make a note on the form.

For the lidar plots, navigate toward the plot center waypoint using Garmin; however, place your plot center exactly at the marked center stake of the original lidar plot. Garmin may not get you exactly to the stake. You may have to look around for it. If you do not find the stake then get as close as you can using Garmin. Note whether you found the stake or not on the data form.

Record the UTM coordinates of your plot center. Tip: press the “mark” key on Garmin as soon as you arrive at plot center. This temporarily captures your coordinates on screen, giving you time to write them down before losing satellite reception. Cancel out of the “mark waypoint” screen after you write down your UTMs.

If plot waypoint is in a clearcut, but it is within 50 meters of trees, then move plot into trees. If plot is in a clearcut that was harvested after the telemetry project ended and it is not within 50 meters of trees, then plot may be skipped. If plot is in a clearcut that was harvested before telemetry project began and it is not within 50 meters of trees, then do plot in clearcut.

If plot waypoint is on a road, then move plot into trees at edge of road. (AH, CC, and CS were not aware of this rule until approximately March 2016. Road plots collected before then were placed in road.)

Name: Initials of data collector

Time: 24hr format

Date: MM/DD/YY

Plot#: Plot number as listed on field maps and as Garmin waypoints. Note, all lidar plots include an underscore (example ##_##).

Slope (degrees): Slope of plot in degrees as measured with a laser relaskop (Criterion RD 1000 electronic BAF scope/dendrometer). Stand at plot center and use laser relaskop to take a degree slope reading upslope and downslope. Add the two readings and divide by two to get an average slope. Enter the average slope on the data form.

Aspect: Use compass to determine which direction the plot slope faces. Enter N, NW, W, SW, S, SE, E or NE on form.

Rat Nest Count: Total number of wood rat nests in plot.

Fuel Loading: Fill in Low, Medium or High (or L, M, H). This is an ocular estimate of fuel load. Refer to figure 1.

Elevation: Elevation in feet as measured by Garmin.

Habitat Type: Dominant habitat type in plot. Examples: Douglas fir (DF), Mixed conifer (MC), Road, Edge, Clearcut, Riparian, Rock Cliff.

Tree Variable Plot Information

Collect the following data for every tree ≥ 10cm in diameter within a variable plot of Basal Area Factor (BAF) 40. If a tree forks below breast height count it as two trees. Use the laser relaskop to identify trees in the BAF 40, measure their DBH (diameter at breast height), and their height. The additional data for each tree will be based on ocular estimation.

Species: The four letter taxonomic code for the tree species (e.g., PSME, ALRU, TSHE). Use capital letters. If unknown, enter "UNKN." Field crews are provided with a list of codes for all plants currently in the database. If a new species is found, add it to the list and create its 4 letter code based on the first two letters of genus and species in the scientific name (e.g., *Pseudotsuga menziesii* = PSME). If that code is already on the list for a different plant, add a number to the end of the code (e.g., PSME2, PSME3, PSME4). Keep careful track of new plant codes. They will be added to the database at the end of the season.

DBH (cm): Record the tree's diameter at breast height (DBH) to the nearest cm using the laser relaskop or DBH tape. Breast height is defined as 4.5 feet or 1.37 meters above the ground. If tree is on a slope, measure DBH from the upslope side of the tree.

Crown Ratio (%): To the nearest 10% increment, estimate and record the % of the height of the tree that is occupied by green (i.e. living) crown. Tip: this is meant to be an ocular estimate; however, it may be helpful to use the laser relaskop to note canopy heights while you are measuring tree height.

Crown Class: Enter **1** for Dominant, **2** for Codominant, **3** for Intermediate, or **4** for Suppressed.

Definitions:

1. Dominant: Trees with crowns extending above the general level of the crown cover and receiving full light from above and partly from the side; larger than the average trees in the stand and with crowns well developed but possibly somewhat crowded on the sides.

2. Codominant: Trees with crowns forming the general level of the crown cover and receiving full light from above but comparatively little from the sides; usually with medium sized crowns more or less crowded on the sides.

3. Intermediate: Trees shorter than those in the two preceding classes, but with crowns either below or extending in to the crown cover framed by the codominant and dominant trees, receiving a little direct light from above, but none from the sides, usually with small crowns considerably crowded on the sides.

4. Suppressed: Trees with crowns entirely below the general level of the crown cover, receiving no direct light either from above or from the sides.

Nest Position: Scan tree for bird or mammal nests. Enter **0** if none are seen. Enter **1** if there is a nest in or against bole of tree. Enter **2** if there is a nest is on a branch.

Nest Size: Enter 0 if no nest is found. Enter 1 if nest is >20 inches in diameter. Enter 2 if nest is <20 inches in diameter.

Defect: Record the most prominent three of the following defects for the tree. If none, leave blank.

C = Cavity(s) in tree – natural or those created by woodpeckers/other animals.
D = Dead top of tree
B = Broken top of tree
F = Forked trunk
K = Conk fungus fruiting bodies are present on tree's trunk or branches

Height (m): To the nearest meter, record the tree height as measured by the laser relaskop and rangefinder. Tips: If measuring a tree upslope of you, use angle compensating settings on the rangefinder to obtain an accurate horizontal distance to the tree. If a tree is leaning, you will obtain a more accurate height measurement if you stand so that the tree is leaning to the right or left of you rather than toward or away from you.

Snag Data Collection (within a 16m radius plot)

Locate all snags within 16m of the plot center (use rangefinder) that have height ≥3m and DBH ≥10cm.

Species: To the best of your ability, determine species and record as for tree species. If unknown, enter UNKN.

DBH (cm): To the nearest cm, measure (with laser relaskop or diameter tape) or estimate and record the DBH.

Height (m): To the nearest m, measure with laser relaskop or estimate and enter the height of the snag.

Decay Class: Refer to figure 2. Enter one of the following categories.
- **I** = Incipient
- **M** = Moderate
- **A** = Advanced

Defect: Record the most prominent three of the following defects for the snag.
- **C** = Cavity
- **B** = Broken top
- **F** = Forked
- **K** = Conk fungus

<u>Shrub and Forb Species Cover Data Collection (within a 16m radius plot)</u>

Record species and percent cover category (see below) for all plants ≤5m tall and within 16m of the plot center (use the rangefinder to establish plot boundaries). You may need to take a brief walk around the plot to locate each species. Tree species may be included if they have live limbs within the shrub layer (i.e., less than 5m in height).

Species: Identify all trees, shrubs, and forbs to species and record their four letter taxonomic code. Enter UNKN for unknown species. Use code UNGR (unknown grass) for all grasses. Also record percent cover for BARE, ROCK, LEAF, and MOSS if present.

Cover %: Within 20% increments, estimate the percent cover of each species and record as code T, 1,2,3,4, or 5 (see below). Tip: For our 16m radius plots, an area of 1% is a circle with a 1.6m radius.
- **T** = Trace (<1%)
- **1** = 1-20%
- **2** = 20-40%
- **3** = 40-60%
- **4** = 60-80%
- **5** = 80-100%

<u>DWD Data Collection (within a 16m radius plot)</u>

Collect Downed Woody Debris (DWD) data at 1 in 10 plots. Try to do an even number of low, medium and high DWD plots. DWD level is the same as fuel load in the general plot information section. Refer to figure 1. Collect the following data on all pieces of DWD that are within 16m of plot center and have a

small end diameter ≥10cm and a length ≥2m. If a DWD piece falls partially in and partially out of the plot, only measure the portion that is within the plot. Most or all of the DWD measurements should be ocular estimates. A fully trained observer should complete DWD plots within about 10 minutes.

Species: To the best of your ability, determine species and record as for tree species. For unknown, enter UNKN.

Length (m): To the nearest meter, estimate the length of the DWD piece. If measurement is required, use the rangefinder to shoot a distance from one end of the DWD piece to the other.

Diameter small (cm): To the nearest cm, estimate the diameter of the small end of the piece of DWD. If measurement is required, use the diameter tape. If the small end of the DWD has a diameter less than 10cm, record "10" and ignore the portion that is smaller than 10cm.

Diameter large (cm): To the nearest cm, estimate the diameter of the large end of the piece of DWD. If measurement is required use the diameter tape.

Decay class: As for snags, enter **I** for incipient, **M** for moderate, or **A** for advanced decay. Refer to figure 3.

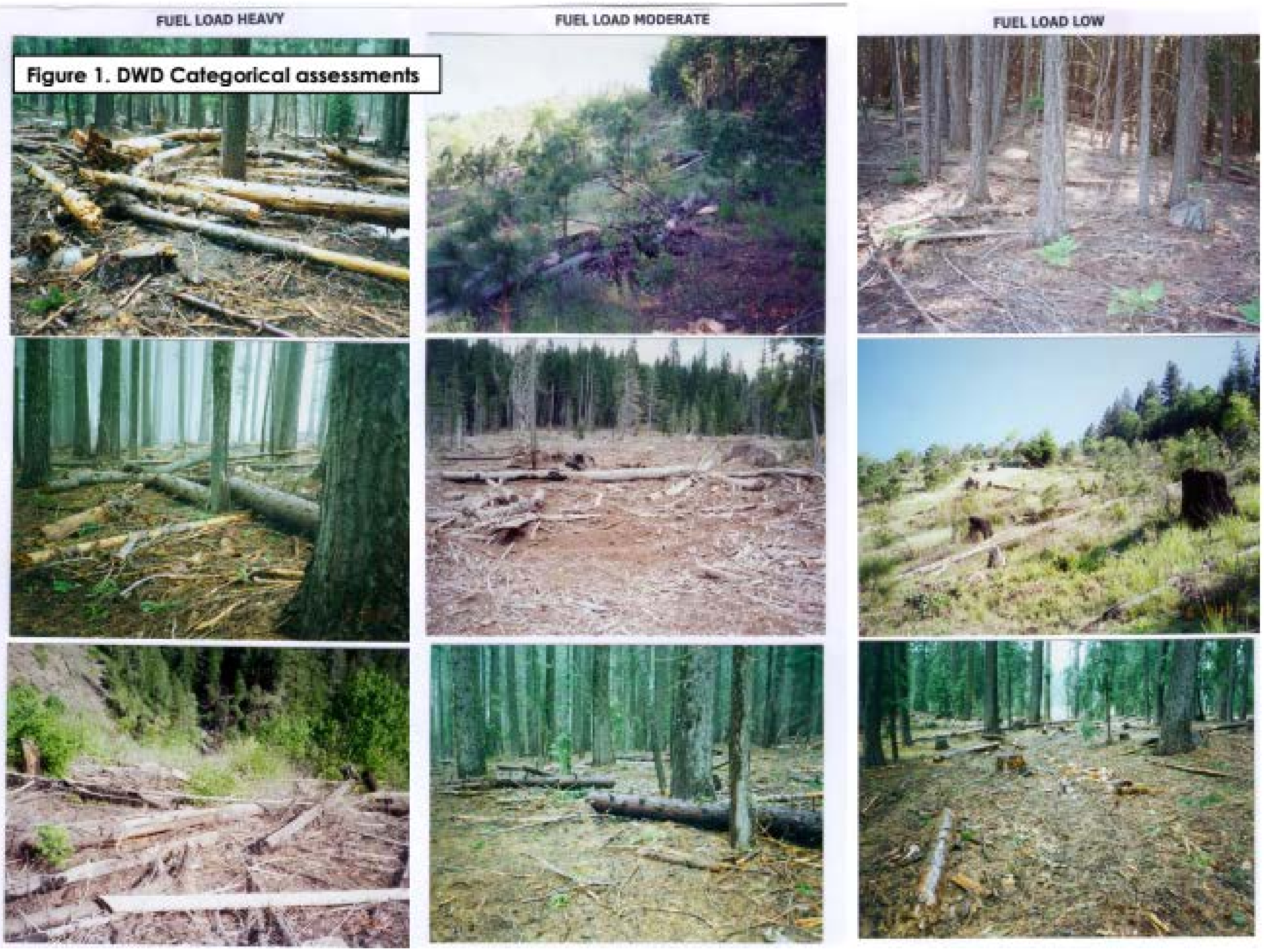

Figure 1. DWD Categorical assessments

Figure 2: Snag Decay Classes

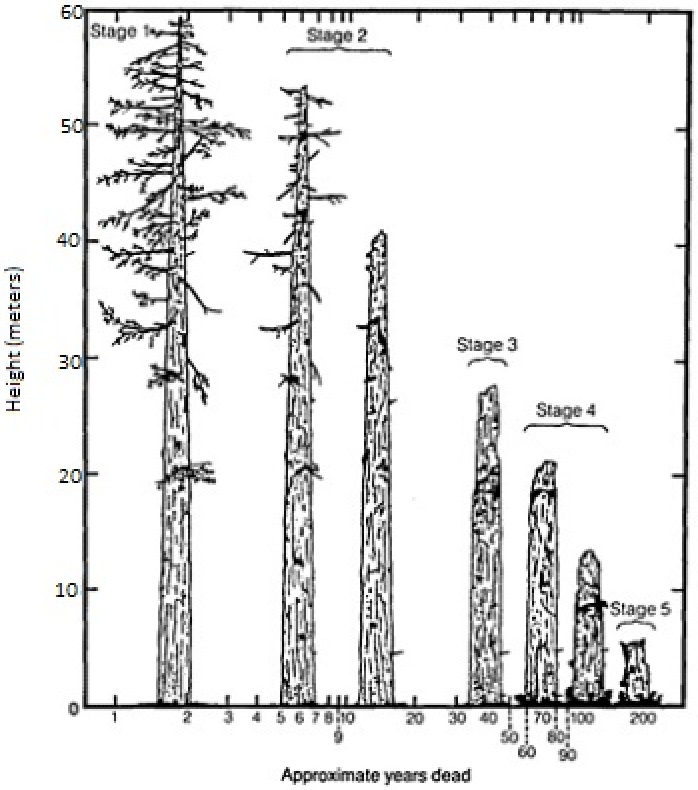

Five stages of deterioration of Douglas-fir snags (adapted from Cline et al. 1980).

Incipient Decay Classes:

1 = Limbs and branches present, top pointed, bark and sapwood intact.

Moderate Decay Classes:

2 = Large limbs and stubs present, upper 10% of bole may have broken off, bark starting to slough, base solid.

3 = Without limbs, stubs present, upper 50% of top broken, bark sloughing, sapwood decaying, and decay onset at base.

Advanced Decay Classes:

4 = Without limbs, few stubs, some bark, sapwood decayed, brown, base decay, 10-20m height.

5 = Without limbs, few if any stubs, sapwood decayed and advanced base decay, less than 10m height.

Figure 3: Downed Woody Debris Decay Classes

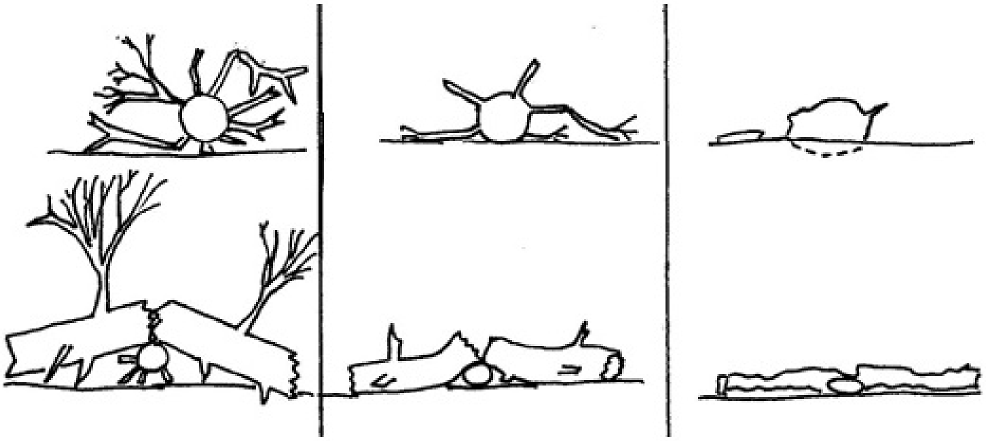

Class 3	Class 4	Class 5
Branches present, not snagging between supports, bark mostly intact, wood intact maybe partly soft.	Large branches only, log sagging or on ground, bark sloughing to absent, sapwood brown blocky pieces.	No branches, no bark, wood sloughing and powdery, log oval shaped with epiphytes.

Incipient Decay Classes:

1 = Tree live. Leaves green.

2 = Fresh killed. Needles or leaves still on.

3 = Recent dead. Bark intact, limbs present, texture mostly sound, shape round, wood original color, elevated on supports (see above).

Moderate Decay Classes:

4 = Intermediate. Bark loose, small limbs missing, sapwood decay present, shape round, wood color starting to fade, all to mostly sagging.

Advanced Decay Classes:

5 = Decomposing. Bark absent, branches stubs loose, round to oval shape, color faded, all of piece on ground.

6 = Decomposed. Wood has deteriorated to punk or tinder (litter).
